# Editorial: Interactions between COVID-19 and malaria

**DOI:** 10.3389/fimmu.2023.1193377

**Published:** 2023-04-13

**Authors:** Stephanie K. Yanow, Maxine Anne Whittaker

**Affiliations:** ^1^ School of Public Health, University of Alberta, Edmonton, AB, Canada; ^2^ College of Public Health, Medical and Veterinary Sciences, James Cook University, Townsville, QLD, Australia

**Keywords:** malaria, COVID-19, SARS-CoV-2, interactions, serology, co-infection, surveillance

As the first wave of SARS-CoV-2 infections spread across the globe there was a noticeable paucity of cases reported from countries in sub-Saharan Africa (SSA). Many theories were proposed to explain the epidemiology of COVID-19 in this region. One relates to population demographics; there is a clear positive correlation between age and risk of infection (1) suggesting the younger age distribution in many countries in SSA could account for fewer cases. Likewise, infection is generally less severe in younger people (2) and the mild symptoms may influence care-seeking behaviours. The case numbers may also reflect limited capacity for SARS-CoV-2 diagnosis. This was particularly relevant prior to the availability of rapid diagnostic tests when diagnosis relied heavily on molecular testing. Together, the population structure and limited diagnostic capacity could contribute to a gross underreporting of cases. An alternative and compelling theory is that the severity and/or risk of infection with SARS-CoV-2 is modulated by malaria. At the same time there were major concerns about the potential impact of COVID-19 and the public health responses to the pandemic upon routine health service delivery, including malaria programmes, and the potential mis-diagnosis of malaria fevers as COVID-19 by providers and communities, who may be concerned about seeking services (3). The six articles within the Research Topic ‘Interactions between COVID-19 and malaria’ address these ideas by considering the potential biological interactions between the two pathogens and more broadly, the public health impact of the pandemic on malaria research and control.

Over 95% of malaria cases occur in SSA with 229 million new cases reported in the year prior to the emergence of SARS-CoV-2 (4). Given this intense force of infection, malaria is considered among the strongest drivers of human evolution and African populations exhibit specific genetic traits that favour resistance to this parasite. One of the most striking examples is selection for the Duffy-null genotype that is strongly associated with resistance to infection by *Plasmodium vivax* (5). This gene encodes the DARC receptor on red blood cells (RBCs), and merozoites from this species rely primarily on binding to DARC to initiate invasion of RBCs. *Plasmodium* can also interact biologically with other pathogens and typically the parasite exacerbates disease, as observed for example during co-infection with HIV (6). In their review article, Konozy et al. explore the biological links between *Plasmodium* and SARS-CoV-2, discuss the evidence that host genetic variation could alter susceptibility to infection and draw parallels between the immunological responses evoked by both pathogens. As these immune pathways are dissected, there are clear commonalities yet little evidence of causality.

One intriguing idea raised by Konozy et al. is that SARS-CoV-2 may infect RBCs *via* receptors, such as CD147/Basigin, that are also used by *P. falciparum* for invasion. This was tested experimentally in the research report by Lopez-Farfan et al. and conclusively disproved. The virus does not bind to CD147 nor does it readily infect RBCs. A further observation was that the remodeling of the RBC membrane by *P. falciparum* does not render cells permissive to SARS-CoV-2 infection. This article provides important evidence that the two pathogens engage distinct human receptors and that co-infection is unlikely. Consistent with these *in vitro* experiments, an epidemiological study by the same authors confirmed a very low rate of co-infection in a study in Burkina Faso (López-Farfán et al.). They tested 998 participants and only 8 were positive for both SARS-CoV-2 and *P. falciparum* (by PCR). All study participants were asymptomatic; thus, it was not possible to glean insight into the effect of malaria co-infection on the severity of SARS-CoV-2 or vice versa. Yet this question was addressed in the study from Mali reported by Woodford et al. They asked whether seasonal malaria affected SARS-CoV-2 seroconversion rates, clinical presentation, and antibody responses. Using a longitudinal study design, they evaluated the effects of recent or current malaria on SARS-CoV-2 infection. They found no evidence to support an effect of malaria infection on antibody responses to the virus or the severity of symptoms at presentation. These data strongly refute the hypothesis that malaria may protect against SARS-CoV-2.

The ability to perform this study in Mali at a time when community-based research was largely impossible is attributed to the research infrastructure already in place to conduct malaria clinical trials. The long-standing collaboration between US and Malian researchers meant that infrastructure established to support malaria trials could be leveraged for sub-studies on COVID-19. As discussed in the opinion article by Woodford et al., research staff responded to the pandemic by expanding their research capacity to study the interactions between malaria and SARS-CoV-2. They also successfully introduced vital engineering controls, promoted staff training and education to enhance safety of staff and participants.

Early in the pandemic when lockdown measures were in full force, a pressing public health concern was that disruptions to essential malaria services would compromise control and surveillance efforts. In effect, 13.4 million additional cases and 63,000 deaths between 2019 and 2021 were attributed to the pandemic (7). This increase in cases raised the alarm for countries on the cusp of malaria elimination that rely on robust surveillance systems to identify isolated cases. In Cabo Verde, the last indigenous case of malaria was reported in 2018 and the country was close to achieving WHO certification of elimination status (based on 3 consecutive years of zero indigenous cases). In the article by DePina et al., the authors examined the impact of COVID-19 restrictions on care-seeking behaviour and the frequency of malaria testing using an interrupted time series analysis. Surprisingly, while there was a 28% decrease in the number of outpatient visits, there was a non-significant 8% *increase* in testing for malaria per 1,000 outpatient visits. As a result of sustained surveillance throughout the pandemic, the country now qualifies for elimination status.

The articles in this Research Topic addressed the specific context in SSA where SARS-CoV-2 emerged in countries already battling malaria. The prospect of negative interactions between the two pathogens, where one could exacerbate the other, would be dire and further compromise malaria control efforts and management of COVID-19. Instead, we learned that the two pathogens seem to be independently pathogenic and seroconversion to SARS-CoV-2 is not impeded by past exposure to malaria. Most promising are the public health lessons on the remarkable resilience of research teams and communities to sustain malaria control efforts and clinical trials under these exceptionally challenging circumstances.

## Author contributions

All authors listed have made a substantial, direct, and intellectual contribution to the work and approved it for publication.
